# Supplementary material for: Social relationships and their associations with affective symptoms of women with breast cancer: A scoping review
Source: PLoS One. 2022 Aug 8;17(8):e0272649. doi: 10.1371/journal.pone.0272649 (PMC9359609; doi:10.1371/journal.pone.0272649)
Supplement: S1 Table — (DOCX) [file pone.0272649.s001.docx]

**Appendix A. Search Strategy**

**Date searched: Feburary 15, 2022**

**Database: Medline (via PubMed)**

| Set # | Terms |
| --- | --- |
| 1 | "Breast Neoplasms"[mesh] OR "breast cancer"[tiab] OR "breast cancers"[tiab] OR "breast tumor"[tiab] OR "breast tumors"[tiab] OR "breast tumours"[tiab] OR "breast tumour"[tiab] OR "breast carcinoma"[tiab] OR "breast carcinomas"[tiab] OR "ductal carcinoma"[tiab] OR "ductal carcinomas"[tiab] OR DCIS[tiab] OR "lobular carcinoma"[tiab] OR "lobular carcinomas"[tiab] |
| 2 | "Affective Symptoms"[MeSH Terms] OR "Affective symptoms"[tiab] OR "Affective symptom"[tiab] OR "Anxiety"[MeSH Terms] OR anxiety[tiab] OR anxious[tiab] OR "depressive disorder"[MeSH Terms] OR "depression"[MeSH Terms] or depression[tiab] or depressed[tiab] OR depressive[tiab] OR "Cognitive Dysfunction"[MeSH Terms] OR "Cognitive Dysfunction"[tiab] OR "Cognitive Dysfunctions"[tiab] OR "Cognitive Impairment"[tiab] OR "Cognitive Impairments"[tiab] OR "Cognitive Decline"[tiab] OR "Neurocognitive Disorder"[tiab] OR "Mental Deterioration"[tiab] OR "cognitive disorder"[tiab] OR "Cognition"[MeSH] OR cognition[tiab] OR "cognitive function"[tiab] or "emotional function"[tiab] OR "Mood Disorders"[MeSH] OR "mood disturbance"[tiab] OR "mood disturbance"[tiab] |
| 3 | "Interpersonal Relations"[Mesh] OR "Social Support"[Mesh] OR "Family"[Mesh] OR family[tiab] OR friendship[tiab] OR friendships[tiab] OR neighbor[tiab] OR neighbors[tiab] OR "co-workers"[tiab] OR coworkers[tiab] OR coworker[tiab] OR "co-worker"[tiab] OR "Friends"[Mesh] OR friend[tiab] OR friends[tiab] OR peer[tiab] OR peers[tiab] OR colleague[tiab] OR colleagues[tiab] |
| 4 | Female[mesh terms] OR female[tiab] or females[tiab] or "Women"[Mesh] OR woman[tiab] or women[tiab] |
| 5 | 1 and 2 and 3 and 4 |
| 6 | NOT (("Adolescent"[Mesh] OR "Child"[Mesh] OR "Infant"[Mesh]) NOT "Adult"[Mesh]) |
| 7 | NOT (Editorial[ptyp] OR Letter[ptyp] OR Comment[ptyp]) NOT (animals[mh] NOT humans[mh]) |

Database: Embase (via Elsevier)

| Set # | Terms |
| --- | --- |
| 1 | 'Breast cancer'/exp OR 'breast cancer':ti,ab OR 'breast cancers':ti,ab OR 'breast tumor':ti,ab OR 'breast tumors':ti,ab OR 'breast tumours':ti,ab OR 'breast tumour':ti,ab OR 'breast carcinoma':ti,ab OR 'breast carcinomas':ti,ab OR 'ductal carcinoma':ti,ab OR 'ductal carcinomas':ti,ab OR DCIS:ti,ab OR 'lobular carcinoma':ti,ab OR 'lobular carcinomas':ti,ab |
| 2 | 'emotional disorder'/exp OR 'Affective symptoms':ti,ab OR 'Affective symptom':ti,ab OR 'Anxiety'/exp OR anxiety:ti,ab OR anxious:ti,ab OR 'depression'/exp or depression:ti,ab or depressed:ti,ab OR depressive:ti,ab OR 'cognitive defect'/exp OR 'Cognitive Dysfunction':ti,ab OR 'Cognitive Dysfunctions':ti,ab OR 'Cognitive Impairment':ti,ab OR 'Cognitive Impairments':ti,ab OR 'Cognitive Decline':ti,ab OR 'Neurocognitive Disorder':ti,ab OR 'Mental Deterioration':ti,ab OR 'cognitive disorder':ti,ab OR 'Cognition'/de OR cognition:ti,ab OR 'cognitive function':ti,ab or 'emotional function':ti,ab OR 'Mood Disorder'/exp OR 'mood disturbance':ti,ab OR 'mood disturbance':ti,ab |
| 3 | 'human Relation'/exp OR 'Social Support'/exp OR 'Family'/exp OR family:ti,ab OR friendship:ti,ab OR friendships:ti,ab OR neighbor:ti,ab OR neighbors:ti,ab OR 'co-workers':ti,ab OR coworkers:ti,ab OR coworker:ti,ab OR 'co-worker':ti,ab OR 'Friend'/exp OR friend:ti,ab OR friends:ti,ab OR peer:ti,ab OR peers:ti,ab OR colleague:ti,ab OR colleagues:ti,ab |
| 4 | 'Female'/exp OR female:ti,ab or females:ti,ab or Women:ti,ab OR woman:ti,ab or women:ti,ab |
| 5 | 1 and 2 and 3 and 4 |
| 6 | AND [adult]/lim AND [humans]/lim |
| 7 | NOT ([editorial]/lim OR [letter]/lim) |

**Database: APA PsycINFO (via EBSCOhost)**

| Set # | Terms |
| --- | --- |
| 1 | DE "Breast Neoplasms" OR TI ("breast cancer" OR "breast cancers" OR "breast tumor" OR "breast tumors" OR "breast tumours" OR "breast tumour" OR "breast carcinoma" OR "breast carcinomas" OR "ductal carcinoma" OR "ductal carcinomas" OR DCIS OR "lobular carcinoma" OR "lobular carcinomas") OR AB ("breast cancer" OR "breast cancers" OR "breast tumor" OR "breast tumors" OR "breast tumours" OR "breast tumour" OR "breast carcinoma" OR "breast carcinomas" OR "ductal carcinoma" OR "ductal carcinomas" OR DCIS OR "lobular carcinoma" OR "lobular carcinomas") |
| 2 | DE "Affective Disorders" OR (DE "Anxiety") OR (DE "Major Depression") OR (DE "Cognitive Impairment") OR (DE "Cognition") OR TI ("Affective symptoms" OR "Affective symptom" OR anxiety OR anxious OR depression OR depressed OR depressive OR "Cognitive Dysfunction" OR "Cognitive Dysfunctions" OR "Cognitive Impairment" OR "Cognitive Impairments" OR "Cognitive Decline" OR "Neurocognitive Disorder" OR "Mental Deterioration" OR "cognitive disorder" OR cognition OR "cognitive function" or "emotional function" OR "mood disturbance" OR "mood disturbance") OR AB ("Affective symptoms" OR "Affective symptom" OR anxiety OR anxious OR depression OR depressed OR depressive OR "Cognitive Dysfunction" OR "Cognitive Dysfunctions" OR "Cognitive Impairment" OR "Cognitive Impairments" OR "Cognitive Decline" OR "Neurocognitive Disorder" OR "Mental Deterioration" OR "cognitive disorder" OR cognition OR "cognitive function" or "emotional function" OR "mood disturbance" OR "mood disturbance") |
| 3 | (DE "Interpersonal Relationships") OR (DE "Social Support") OR (DE "Family") OR (DE "Friendship") OR TI (family OR friendship OR friendships OR neighbor OR neighbors OR "co-workers" OR coworkers OR coworker OR "co-worker" OR friend OR friends OR peer OR peers OR colleague OR colleagues) OR AB (family OR friendship OR friendships OR neighbor OR neighbors OR "co-workers" OR coworkers OR coworker OR "co-worker" OR friend OR friends OR peer OR peers OR colleague OR colleagues) |
| 4 | DE "Human Females" OR TI (female or females OR woman or women) OR AB (female or females OR woman or women) |
| 5 | 1 and 2 and 3 and 4 |
| 6 | Limiters - Age Groups: Adulthood (18 yrs & older); Population Group: Human |
| 7 | NOT PZ (Editorial OR Letter OR Comment/Reply) |

Database (including vendor/platform): Web of Science Core Collection Editions = A&HCI, BKCI-SSH, BKCI-S, CCR-EXPANDED, ESCI, IC, CPCI-SSH, CPCI-S, SCI-EXPANDED, SSCI (via Clarivate)

| Set # | Terms |
| --- | --- |
| 1 | TS=("breast cancer" OR "breast cancers" OR "breast tumor" OR "breast tumors" OR "breast tumours" OR "breast tumour" OR "breast carcinoma" OR "breast carcinomas" OR "ductal carcinoma" OR "ductal carcinomas" OR DCIS OR "lobular carcinoma" OR "lobular carcinomas") |
| 2 | TS=("Affective symptoms" OR "Affective symptom" OR anxiety OR anxious OR depression or depressed OR depressive OR "Cognitive Dysfunction" OR "Cognitive Dysfunctions" OR "Cognitive Impairment" OR "Cognitive Impairments" OR "Cognitive Decline" OR "Neurocognitive Disorder" OR "Mental Deterioration" OR "cognitive disorder" OR cognition OR "cognitive function" or "emotional function" OR "mood disturbance" OR "mood disturbance") |
| 3 | TS=(family OR friendship OR friendships OR neighbor OR neighbors OR "co-workers" OR coworkers OR coworker OR "co-worker" OR friend OR friends OR peer OR peers OR colleague OR colleagues) |
| 4 | TS=(female or females OR woman or women) |
| 5 | 1 and 2 and 3 and 4 |
| 6 | **Refined by:** [excluding] **DOCUMENT TYPES:** ( EDITORIAL MATERIAL ) |
