# Supplementary material for: Social relationships and their associations with affective symptoms of women with breast cancer: A scoping review
Source: PLoS One. 2022 Aug 8;17(8):e0272649. doi: 10.1371/journal.pone.0272649 (PMC9359609; doi:10.1371/journal.pone.0272649)
Supplement: Attachment — Submitted filename: Authors Response_0227_2022.docx [file pone.0272649.s003.docx]

February 27, 2022

We are grateful for the careful review made by the reviewers. Below, you will find an itemized account of our responses to the reviewer’s feedback. Additionally, we track changed texts in the manuscript where revisions were made to facilitate your review. We Sincerely hope that our changes satisfy the request for revision. Thank you.

| **Reviewer’s Comment** | **Authors’ Response** |
| --- | --- |
| In the case of your submission, we are concerned that the literature review was completed in September 2020. Systematic and scoping reviews should provide an up to date overview of the research topic covered. Since the literature search was completed over a year ago, we are concerned that this study does not meet this requirement.  If you are able to address this concern through revisions to your manuscript, we may be willing to consider a resubmission for your work. The revised manuscript should be submitted as a new submission to PLOS ONE and accompanied by a cover letter that refers to the original submission number and outlines details of how the revisions have been completed.  Please note that further consideration is dependent on the submission of a manuscript that addresses the above concern. We will carefully review your manuscript upon resubmission, so please ensure that your revision is thorough. | Thanks for the comment. We updated our literature search and this updated manuscript now includes all the relevant literature available on MEDLINE (PubMed), Embase (Elsevier), PsycINFO (EBSCOhost), and Web of Science (Clarivate) published on or before February 15, 2022. |
