# Supplementary material for: Social relationships and their associations with affective symptoms of women with breast cancer: A scoping review
Source: PLoS One. 2022 Aug 8;17(8):e0272649. doi: 10.1371/journal.pone.0272649 (PMC9359609; doi:10.1371/journal.pone.0272649)
Supplement: Attachment — Submitted filename: Authors Response_0614_2022.docx [file pone.0272649.s004.docx]

June 14, 2022

We are grateful for the careful review made by the reviewers. Below, you will find an itemized account of our responses to the reviewers’ feedback. Additionally, we highlighted sections in the manuscript where revisions were made to facilitate your review. We believe that we have addressed all of the concerns and that these changes have strengthen the manuscript. Thank you for your consideration.

| **Editor’s Comments** | **Authors’ Response** |
| --- | --- |
| 1. Expand the introduction to clearly explain/define what is meant by affective symptoms. Throughout the paper, depression and anxiety are primarily discussed, but other affective symptoms and measures are included in the review. Clear operationalization and justification of what does and does not constitute affective symptoms is necessary. It would also help to better situate this review in the existing literature. Why is important to examine affective symptoms in breast cancer populations? | Done. Included definition of affective symptoms (Lines 72-76) and explained significance of studying affective-cognitive symptoms in breast cancer population (Lines 76-79). |
| 2. Similarly, the discussion of social relationships in the introduction should be expanded. A more representative discussion of the literature linking social relationships to affective symptoms, particularly your definition of affective symptoms, is necessary. In your review, you emphasize the distinction between functional and structural components of social relationships. Why is this distinction important? What evidence is there to suggest that certain components of social relationships are more important than others? To prepare the reader for the review, it would be helpful to introduce and define different components of social relationships (e.g., social integration, social support, functioning), particularly those components covered in the review. | Done. Revised introduction to show that it is important to understand both aspects of social relationships and their link with affective-cognitive symptoms because this comprehensive understanding will be critical for risk identification, prevention, and treatment strategies for those with these symptoms (Lines 106-108).  Included definition of structural and functional aspects of social relationships (Lines 90-93) |
| 3. In the inclusion/exclusion criteria, provide detailed operational definitions for affective symptoms and social relationships. What measures were acceptable versus not? As is, the review covers a broad range of affective symptoms and social relationship measures, with some variables or concepts only represented once. It may be worth refining your operational definitions/inclusion criteria to have a more focused review with clearer implications. | Done. Revised Table 1. |
| 4. I take it articles were limited to peer-reviewed papers. Please specify. | Done. Please refer to Table 1. |
| 5. Did any of the papers involving secondary data analysis use datasets from any of the other articles included in the review? Is there independence of findings? | Yes, Hasson-Ohayon 2014 and 2010 used the same datasets (Lines 195-196). |
| 6. In the discussion, it would be helpful to elaborate on potential methodological or sample differences that might explain inconsistent findings. | Done (Lines 382-393) |
| 7. The practical implications section is pretty generic. I recommend developing more nuanced suggestions based on the findings of the review and highlighting unique components of social relationships. | Done (Lines 407-433) |
| 8. Throughout the paper, causal language is used regarding the relationship between social relationships and affective symptoms. As most of the literature reviewed is correlational, causal inference is not possible. Tighten up the language to more accurately discuss the association. | Done. |
| **Reviewer’s Comments** | **Authors’ Response** |
| 1. Is the manuscript technically sound, and do the data support the conclusions?  The manuscript must describe a technically sound piece of scientific research with data that supports the conclusions. Experiments must have been conducted rigorously, with appropriate controls, replication, and sample sizes. The conclusions must be drawn appropriately based on the data presented.  Reviewer #1: Partly | Done. Reviewed and revised the entire manuscript. |
| 2. In the Abstract (lines 62-64) section, the authors state “Of seventy included studies, none of them focused on cognitive symptoms of breast cancer patients; thus, in this review, we focused on only the affective symptoms of breast cancer patients and their association with patients’ both aspects of social relationships.” – this is not a main finding. The main point is that social support/network may be associated with better affective symptom outcomes. Please rewrite the result. | Done. (Lines 49-51) |
| 3. How about focusing on writing the association between social support and affective symptoms throughout and briefly mentioning social relationships with cognitive symptoms in the limitation section as no studies on cognitive symptoms were included? | Thanks for suggestions. As suggested, I revised the introduction to show that this paper focused on affective symptoms. Also, in the limitation section, I indicated that none of included studies discussed cognitive symptoms (Lines 435-443).  Because we used cognitive symptoms as search terms, I think it would make readers somewhat confused if I don’t mention cognitive symptoms at all in the Introduction. For this reason, I used affective-cognitive symptoms throughout the Introduction. |
| 4. It would be helpful to add a meta-analysis of the associations between social support and affective symptoms to estimate how strong an association is between two variables given a great volume of the studies included in the review. | Thanks for suggestion. The purpose of our scoping review was to map the literature on social relationships associated with affective-cognitive symptoms of breast cancer patients. Because Scoping Reviews include a wide range of study types, the data is not homogenous enough to effectively conduct a meta-analysis.”  See the PRISMA extension for Scoping Reviews (PRISMA-ScR):  “Item 13 (Not Applicable): Summary Measures - This item from the original PRISMA is not applicable for scoping reviews because a meta-analysis is not done (that is, summary measures are not relevant).”  Which you can review here:  Tricco, AC, Lillie, E, Zarin, W, O'Brien, KK, Colquhoun, H, Levac, D, Moher, D, Peters, MD, Horsley, T, Weeks, L, Hempel, S et al. PRISMA extension for scoping reviews (PRISMA-ScR): checklist and explanation. Ann Intern Med. 2018,169(7):467-473. [doi:10.7326/M18-0850.](https://urldefense.com/v3/__http:/annals.org/aim/fullarticle/2700389/prisma-extension-scoping-reviews-prisma-scr-checklist-explanation__;!!KGKeukY!xWKL4gp9ijwW0oq8vO4kbRm4Ly4J_U-Hdsj8sw24Pj9ZdFyfD4jguycAlgIU8v9FBcIP4bwZdp6IO0-ZIhS-oQv50jYbVnB4$) |
| 5. The authors may include the study quality evaluation for each study in the table. | Thanks for suggestion.  From the PRISMA-Scoping Guidelines document, Item 12: Explanation and Elaboration- “A key difference between scoping reviews and systematic reviews is that the former is generally conducted to provide an overview of the existing evidence regardless of methodological quality or risk of bias.”  Therefore, the included sources of evidence are typically not critically appraised for scoping reviews.”  Which you can review here:  Tricco, AC, Lillie, E, Zarin, W, O'Brien, KK, Colquhoun, H, Levac, D, Moher, D, Peters, MD, Horsley, T, Weeks, L, Hempel, S et al. PRISMA extension for scoping reviews (PRISMA-ScR): checklist and explanation. Ann Intern Med. 2018,169(7):467-473. [doi:10.7326/M18-0850.](https://urldefense.com/v3/__http:/annals.org/aim/fullarticle/2700389/prisma-extension-scoping-reviews-prisma-scr-checklist-explanation__;!!KGKeukY!xWKL4gp9ijwW0oq8vO4kbRm4Ly4J_U-Hdsj8sw24Pj9ZdFyfD4jguycAlgIU8v9FBcIP4bwZdp6IO0-ZIhS-oQv50jYbVnB4$) |
| 6. It seems that intervention articles (e.g., mindfulness) were also included in the analysis. Please clarify the inclusion criteria in the method section | Done. Please refer to Table 1. |
| 7. Social relationships with affective symptoms would differ depending on the cancer treatment trajectory (pre-, during, and post-treatment). Survivors would need more social relationships/support before or during cancer treatment than those after cancer treatment. It would be helpful to take a further look at how social relationships with affective symptoms may differ over the treatment trajectory. | Done (Lines 346-354). |
| 8. The discussion section simply addressed the functional and structural social relationships. As the authors indicated in Table 3, several variables seem to influence the association between social relationships and affective symptoms, such as desirability, cancer stage, comorbidity, sociodemographics (e.g., income, race, employment), lifestyle, etc. It would be helpful to address how mediating or moderating factors play a role in affecting the influence of social support on affective symptoms | Done (Lines 355-369) |
| **Minor comments:** 1. Please do not use the term "Caucasian." It is a perjorative term with connotations of racial supremacy. The correct term is "White."  2. Line 127: We reported the findings using…  3. Please change PRISMA-Sc to PRISMA-ScR.  4. Table 1: In the “exclusion criteria” section, please remove periods (.)  5. Line 229-230: “In six studies” was written twice.  6. Line 248: Please use either one (In contrast/However).  7. Please abbreviate terms upon first use – e.g., Randomized controlled trials (RCT)  8. Table 3: hose mates -> house mates  9. Please keep consistency between NR or “not reported” in the table.  10. An editor may help with the writing of the manuscript. | 1. Done. Replaced “Caucasian” with “White”  2. Done (Line 120).  3. Done.  4. Table 1 was revised  5. Thanks for suggestion. I chose “However”.  6. Done. Changed to “However”  7. Done.  8. Done.  9. Done.  10. Thanks for suggestion. Yes, this paper was reviewed by an academic editor. |
| **Journal Requirement** | **Authors’ Response** |
| 1. Please ensure that your manuscript meets PLOS ONE's style requirements, including those for file naming. The PLOS ONE style templates can be found at | Done. |
| 2. We note that you have stated that you will provide repository information for your data at acceptance. Should your manuscript be accepted for publication, we will hold it until you provide the relevant accession numbers or DOIs necessary to access your data. If you wish to make changes to your Data Availability statement, please describe these changes in your cover letter and we will update your Data Availability statement to reflect the information you provide | Done. (DOI 10.17605/OSF.IO/F3ME9; <https://osf.io/f3me9/>) |
| 3. In your Data Availability statement, you have not specified where the minimal data set underlying the results described in your manuscript can be found. PLOS defines a study's minimal data set as the underlying data used to reach the conclusions drawn in the manuscript and any additional data required to replicate the reported study findings in their entirety. All PLOS journals require that the minimal data set be made fully available. For more information about our data policy  "Upon re-submitting your revised manuscript, please upload your study’s minimal underlying data set as either Supporting Information files or to a stable, public repository and include the relevant URLs, DOIs, or accession numbers within your revised cover letter. For a list of acceptable repositories, please see [http://journals.plos.org/plosone/s/data-availability#loc-recommended-repositories](https://urldefense.com/v3/__http:/journals.plos.org/plosone/s/data-availability*loc-recommended-repositories__;Iw!!KGKeukY!y3uAtLa6bK7V2sMVxhjXhtMd6eyrSwEqwBIfhHHg7Zd7h-ERAu0u7MKCy1q5jq6e0COjwFb9UihSIreFlVx5$). Any potentially identifying patient information must be fully anonymized. | Done. (DOI 10.17605/OSF.IO/F3ME9; <https://osf.io/f3me9/>) |
